# Long roads

**DOI:** 10.1093/jhps/hnae001

**Published:** 2024-02-28

**Authors:** Richard E Field

**Affiliations:** Editor-in-Chief, Journal of Hip Preservation Surgery

It is nearly five decades since I was accepted to study Medicine at the Westminster Medical School [1], in London. The hospital closed thirty years ago and was converted into luxury apartments. I sometimes pass the building and think wistfully of my formative years and the many wonderful role models who inspired my love of medical practice. A few days ago, a London black cab drove me past the nearby Westminster Children’s hospital where, as Professor Paul Aichroth’s resident, I assisted his Salter and proximal femoral de-rotation osteotomies. In the three-and-a-half decades since those operations, I have treated some of his patients and invariably marvel at the results of his handiwork. Almost always, it is the contralateral, untreated hip that has become symptomatic.

My teachers relied on their experience, surgical expertise and the occasional intra-operative x-ray image to guide their corrections. In this issue, we present a paper, from Dr Tachibana and his colleagues, in Japan, that analyses survivorship following periacetabular osteotomy for hip dysplasia based on three-dimensional acetabular coverage [2]. The authors have used pre- and post-operative CT scans to quantify acetabular coverage and assess how change in antero-superior, superior and postero-superior coverage influences the likelihood of progression to arthritis. While the paper does not describe any novel surgical technique, it does demonstrate the need for pre-operative, three-dimensional modelling and strengthens the case for the development of surgical strategies to ensure the level of geometric accuracy that arthroplasty surgeons now achieve with robotic-assisted placement of their implants [3].

Medical innovations are often adopted more slowly than we imagine. It is more than thirty years since Richard Villar introduced me to hip arthroscopy. The technique is no longer viewed as an ‘operation looking for an indication’ with telescopic procedures about the hip now accepted as providing the least intrusive means to address both intra and extra-articular pathology [4]. While Japanese surgeons played a pivotal role in the early development of arthroscopic surgery [5], the number of arthroscopic interventions for hip-related problems has grown relatively slowly in Japan. In a second paper from Japan, Dr Fukushima and his colleagues provide a fascinating insight into the different hip preservation procedures undertaken in a country where hip dysplasia is the most common challenge facing hip preservation surgeons [6]. The paper highlights the potential for adverse outcomes when arthroscopic interventions are undertaken in the presence of hip instability and provides a fascinating insight into the drivers for hip preservation surgery in different geographic regions.

One of the highlights of my training was to assist a Dunn procedure [7, 8] to reposition a slipped femoral capital epiphysis. This was a few years before the Bernese strategies to minimize retinacular vessel disturbance were published [9, 10]. The operation was performed by Richard Todd, one of Denis Dunn’s trainees, in the hospital where Dunn had pioneered the procedure. I can still visualize the elegant exposure and preservation of the retinacular vessels and still remember wondering whether the femoral neck shortening would have long-term consequences. In this issue, a new extra-articular strategy is reported by the McMaster group [11]. The technique utilizes both Imhauser [12, 13] and Morscher [14, 15] osteotomies and uses computer-aided planning both to ensure optimal hip geometry and the avoidance of limb shortening. While the technique is only described for two patients, it may prove to be a further milestone in the long journey to address this most challenging clinical condition.

The three highlighted papers in this issue reflect both the diverse challenges of hip preservation surgery and the fact that none of these challenges has been solved over a single surgical career. As with any surgical journey, opportunities to reflect on current knowledge can be helpful. With this in mind, we have invited Prof Paul Beaulé and Dr John Christoferetti to guest edit two special JHPS issues in 2024. Prof Beaulé will be compiling a special issue on dissatisfaction after hip arthroscopy and Dr Christoforetti will be compiling a special issue on groin-related conditions. We believe that overviews of the state of current knowledge on these topics will be valuable to the hip preservation community and we will be soliciting manuscripts for both issues over the next few months. If you would like to be involved in the preparation of these special issues please let us know at jhps.editorialoffice@oup.com.
