# Correction to: Long Roads

**DOI:** 10.1093/jhps/hnae025

**Published:** 2024-06-26

This is a correction to: Richard E Field, Long roads, *Journal of Hip Preservation Surgery*, Volume 11, Issue 1, January 2024, Pages 1–2, https://doi.org/10.1093/jhps/hnae001

The following changes have been made to the originally published manuscript. Sections **Data availability, CONFLICT OF INTEREST STATEMENT** and **Funding**, and their contents, have been removed from the article.

